# Electrophysiological Changes Between Patients With Suicidal Ideation and Suicide Attempts: An Event-Related Potential Study

**DOI:** 10.3389/fpsyt.2022.900724

**Published:** 2022-05-20

**Authors:** Sung Hoon Yoon, Se-Hoon Shim, Ji Sun Kim

**Affiliations:** Department of Psychiatry, Soonchunhyang University Cheonan Hospital, Cheonan, South Korea

**Keywords:** impulsivity, suicidal ideation, suicide attempt, ERP, ACSS

## Abstract

**Introduction:**

Inhibitory control is regarded as an important ability related to the transition from suicidal ideation to suicide attempts. In event-related potential, patients with dysfunction of inhibitory control demonstrate a reduction in the no-go amplitude. This study aimed to determine the association between the no-go event-related potential component and suicidal behaviors among suicide attempters and ideators who never attempted suicide.

**Methods:**

Overall, 150 patients who visited the emergency room by suicide attempts or patients who visited the psychiatric department with suicidal ideation were recruited and instructed to perform a go/no-go task during electroencephalography recording. The Beck Depression Inventory, Beck Anxiety Inventory, Barratt Impulsivity Scale, Difficulties in Emotional Regulation Scale, and Acquired Capability for Suicide Scale were used. Individuals were divided into two groups: those with suicide attempt group) and with suicidal ideation (SI group) without SA. The psychological characteristics and event-related potentials of the two groups were compared. Correlation analyses were conducted to test the association between the clinical characteristics and event-related potentials.

**Results:**

The SA group had significantly decreased no-go P3 amplitudes at all electrodes compared to the SI group. In the correlation analysis between the clinical measurements and event-related potentials in all the participants, no-go P3 amplitudes in whole electrode sites were negatively correlated with the scores of the acquired capability for the suicide scale.

**Conclusions:**

This study revealed that suicide attempters have dysfunction in controlling inhibition compared to suicide ideators reflected in the no-go P3. Our findings suggested that no-go P3 can be a biomarker associated suicide attempts in suicide ideators.

## Introduction

In Korea, almost 14,000 people died by suicide in 2020, with a suicide rate of 25.7 per 100,000 people, indicating that suicide attempts (SA) are the fifth most common cause of death (1). According to World Health Organization statistics, more than 700,000 people die by suicide yearly, which is one person every 40 s (2). Therefore, the interest in preventive measures to diminish suicide risk is constantly increasing. Although suicide is recognized as a social problem and a tool is needed to find correlated the risk of suicide attempts, scientific research results that distinguish between those who have only suicidal ideation (SI) and those who have a history of SA are limited (3, 4). Studies conducted on suicide attempters also have a long time gap between actual SA and research points, making it difficult to verify the temporal causal relationship of the variables significantly associated with SA (5). Moreover, since most of the current studies are limited to specific age groups, such as children and the elderly, studies including various age groups are needed.

Inhibitory control is a facet of prefrontal cortex-driven cognitive control, which may be useful for understanding the progression from SI to SA (6, 7). Numerous theoretical perspectives suggest that defects in inhibitory control may increase the possibility that SI is developed into SA by way of difficulty to control impulsive behavior (i.e., enacting a suicide plan) in an acute suicidal crisis (6, 8). In this context, Venables et al. (9) suggested that a decreased response inhibition and increased threat sensitivity (fear/fearlessness) each independently predicted suicide risk and that both could be stronger biological factors in predicting suicide. Ponsoni et al. (10) found that in patients with mood disorders, those with suicide history had more impulsivity than those without on a self-reported scale. Based on these studies, we attempted to compare impulsivity in two groups to evaluate the effect of impulsivity on the transition from SI to SA.

Few studies have examined neural activity and SA history during an inhibitory control tasks (6, 7, 11, 12). One previous study has revealed that SA had abnormal spontaneous neural activity during the resting state, and decreased activity in the left superior frontal gyrus and left middle frontal gyrus was associated with increased impulsivity in SA on functional MRI (fMRI) (13). However, fMRI cannot reveal differences in neural regions during inhibitory tasks because fMRI lacks temporal resolution (12). From this point of view, event-related potential (ERP) is a methodology that has high temporal resolution (14). A recent meta-analysis has reported that the ERP obtained by performing the go/no-go task was related to inhibitory control (15). Go/no-go tasks are generally used to measure inhibitory control, in which participants required to frequently press a button (i.e., go trials) and infrequently withhold this response (i.e., no-go trials). At least two rapid, successive neural processes comprise inhibitory control: (1) detecting the need for inhibition (i.e., conflict detection) (14–16) and (2) suppressing inappropriate behavior (i.e., motor inhibition) (17, 18). In particular, no-go N2 and no-go P3, which are derived from the no-go task, are known to be highly related to inhibitory control (16). For instance, during go/no-go inhibition tasks, individuals must engage in conflict detection to determine when a no-go stimulus is presented. This conflict detection signal is reflected in the ERP component, which is a frontal negative deflection peaking ~200 ms following no-go stimuli, called no-go N2. If no-go N2 has a more negative amplitude, more attention may be needed to control inhibition (17, 18). By contrast, the P3 component derived from a go/no-go task is a frontocentrally maximal component peaking approximately 300 ms (19) which reflects greater cognitive resources devoted to inhibiting motor responses (20). A previous study has reported that the N2 and P3 components in the no-go task demonstrated changes such as decreased amplitude in patients with poor impulse control, such as trichotillomania, antisocial personality disorder, or attention deficit hyperactivity disorder (21–23). Hence, neurophysiological changes, such as a reduction in the amplitude of no-go N2 or P3, cause difficulties in inhibitory control and poor impulse control. Additionally, a recent study reported that patients with SI showed a lower P3 amplitude than normal controls, indicating that the SI group had more impulsive traits (24). Given that SI is one of the strongest predictors of SA, patients in the SA group are more likely to have a significantly decreased impulse control than those in the SI group who have never attempted suicide. Decreased impulse control can result in changes in biomarkers such as a decreased amplitude of the no-go ERP component.

Despite a plausible relationship between no-go ERP and SA, the relationship between no-go ERP amplitude and SA has not been investigated to date (25). Although one previous study has evaluated the association between no-go ERP and SA, the study had a small sample size of patients taking psychiatric medications. Some studies have shown a greater decrease in p300 in depressive patients with SA rather than those without history of SA, but they all had a small sample size (26, 27). Chen et al. (28) suggested that the SA group had higher P3 amplitude than the non-SA group; however, they did not exclude patients receiving medication which can affect the ERP component. Additionally, these studies explored the relationship between *p3*00 and impulsivity, but it is difficult to determine whether response inhibition and conflict detection were accurately evaluated because the studies did not use the go/no-go trial. Therefore, studies using larger sample sizes of the adult drug naïve population are urgently needed to evaluate the clinical correlation of no-go ERP with SA. This study aimed to determine the association between the no-go ERP component and suicidal behaviors in individuals with SA and SI who have never attempted suicide. Additionally, by measuring impulsivity and other psychiatric symptoms in the two groups, we examined the correlations between psychiatric symptoms, such as impulsivity, suicide capability, and ERP changes. We hypothesized that clinical characteristics, such as impulsivity, and suicide capability, and no-go ERP, differ between patients with depression with SA and those with SI.

## Materials and Methods

### Participants

Initially, 152 participants (76 SI and 76 SA) were enrolled in this study between September 2017 and January 2020. However, two patients with SA dropped out because they declared that they no longer wanted to participate in this study. Thus, a total of 150 participants (76 SI and 74 SA) were enrolled in this study. Patients with SA were referred after being admitted to the internal medicine department due to suicide attempts, which required medical intervention through an emergency room. Determining the minimum sample size was difficult as there are no previous papers that directly compared the correlation between impulsivity and no-go p3 in SI and SA. Therefore, Cohen's f—which is widely used in effect size calculations, as described in of “The Incorporation of Effect Size in Information Technology, Learning, and Performance Research”—was used (29). With this effect size, the minimum sample size was calculated as 132 participants. Therefore, a sample size of 150 was determined to be adequate. The estimates were calculated using G^*^Power software, version 3.1.9.7 (30). Patients with SI were defined as those who did not have a history of suicide but had SI with other depressive symptoms and visited the Department of Psychiatry at Soonchunhyang University Cheonan Hospital. Additionally, patients with depression with SI were enrolled after a psychiatric interview to confirm their SI and intent, using the Beck Scale for Suicide Ideation (31). The participants were interviewed using the Korean version of the MINI International Neuropsychiatric Interview. Participants with psychotic disorders, intellectual disabilities, neurological or severe medical diseases, a history of alcohol or substance abuse/dependence, head trauma, or pregnant women were excluded from the study by screening interviews. In the case of SA, electroencephalography (EEG) was performed within 1 week of suicide attempts, and all subjects were drug naïve. In the case of SI, outpatients who did not take psychiatric drugs were recruited and EEG was conducted on their first visit. All the participants were aged between 19 and 60 years, with their right hand as the dominant hand, and had the normal hearing ability. All the participants provided written informed consent. This study was approved by the Institutional Review Board and Ethics Committee of Soonchunhyang University Cheonan Hospital, and all experimental protocols were approved by this committee (2017-06-035). Participants were informed that they could withdraw their consent at any time and the study was performed in line with approved guidelines. Informed consent was obtained from all study participants and all consent forms were completed by the participants themselves.

### Clinical Measures

All the participants were assessed for psychiatric symptoms, such as depressive mood, anxiety, impulsivity, and emotional dysregulation. To assess the abovementioned clinical characteristics, the Korean versions of the Beck Depression Inventory (BDI), Beck Anxiety Inventory (BAI), Barratt Impulsiveness Scale-11-Revised (K-BIS-11-R), and Difficulties in Emotion Regulation Scale (DERS) were used. The BDI is a self-report examination developed to measure depression. The BDI consists of 21 items, with each item's score ranging from 0 to 3, and the total score ranging from 0 to 63 (32). Higher scores are positively correlated with a severe level of depression. The BAI consists of 21 items, with each item's score ranging from 0 to 3, and the total score ranging from 0 to 63 (33). Higher scores were positively correlated with higher anxiety levels. The BIS is a self-report questionnaire used to evaluate impulsivity and consists of three factors: attention impulsivity, motor impulsivity, and non-planning impulsivity (34). The K-BIS-11-R, which translated this scale into Korean, has proven its reliability and validity for impulsive evaluation. We also used the DERS to evaluate emotion regulation ability. The DERS is a self-reporting tool that consists of a Likert scale 36 items, with each item's score ranging from 1 to 5 (35). Finally, we used the Acquired Capability for Suicide Scale (ACSS) to evaluate suicide capability. The ACSS is a 20-item self-report measure of the extent to which individuals perceive themselves as capable of performing or being exposed to potentially dangerous or fatal situations, including suicide, with scores ranging from 0 to 4 (36).

### EEG Data Acquisition and Analysis

During EEG task, all participants were seated approximately 60 cm away from the computer screen in a sound-attenuated EEG room. EEG signal was acquired using a NeuroScan SynAmps amplifier (Compumedicus USA, E1 Paso, TX, USA) with 64 Ag/AgCl electrodes mounted on a QuikCap. Electrodes were placed as frontal (Fz), central (Cz), parietal (Pz), and an earth electrode was placed frontoparietal (FPz), according to the extended 10–20 placement scheme. To monitor eye movement, an electrode was placed infraorbitally, and the reference electrodes were placed at the mastoid. The impedance was kept below 10 kΩ. All data were processed with a 0.1–100 Hz band pass filter and sampled at 1,000 Hz.

EEG data were processed by CURRY 8 (Compumedics USA, Charlotte, NC, USA). Gross artifacts, such as artifacts caused by movement, were rejected through visual inspection by a trained person with no prior information regarding origin of data. Artifacts related to eye movement were removed using the mathematical procedure of the preprocessing software. Data were filtered using a (1.0–30) Hz band pass filter and epoched from 500 ms pre-stimulus to 900 ms post-stimulus. These epochs were subtracted from the average value of the pre-stimulus interval for the baseline correction. If any remaining epochs continued to have significant physiological artifacts (amplitude exceeding ± 75 μV) at any of the 62 electrode sites, they were excluded from further analysis. Only artifact-free epochs were averaged across trials and participants for ERP analysis. Based on previous studies that no-go ERP reflected behavioral inhibition (16, 20, 37), this study included no-go trials in the ERP analysis.

### Behavioral Task Paradigm

We applied the “oddball paradigm” of auditory stimulation as stimuli for the go/no-go task. ERPs were elicited binaurally using headphones. The participants were instructed to press the spacebar as accurately and quickly as possible when the target tone appeared, and not to respond when the non-target tone appeared. Overall, 400 trials were conducted, which consisted of go (85% probability) and no-go (15% probability) conditions. The target tone (no-go) was 1,500 Hz, and the non-target tone (go) was 1,000 Hz, with a 1,500 ms interval before the next trial. These stimuli were generated using the E-Prime software (Psychology Software Tools, Pittsburgh, PA, USA). In the go/no-go condition, N200 (the most negative peak between 150 and 350 ms after stimulus onset) and P300 (the most positive peak between 250 and 500 ms after stimulus onset) were investigated at the frontal (Fz), fronto-central (FCz), central (Cz), and parietal (Pz) electrodes. The time window assumed during the trials was based on previous studies (38). To accumulate behavioral data, go accuracy, no-go accuracy, and reaction time were calculated based on the data from the E-Prime software. No-go accuracy was calculated to determine the false alarm rate of responses to non-target stimuli.

### Statistical Analysis

To compare differences in demographic data, clinical measurements, and behavioral task data, both groups were compared using the chi-square test for discontinuous variables. For continuous variables, after verifying whether the normality assumption was satisfied by the Shapiro–Wilk test, Mann–Whitney *U* test, or independent *t*-test was used. N2 and P3 amplitudes and latencies of patients were initially evaluated using repeated measures analysis of variance (ANOVA) with electrodes (Fz, FCz, Cz, and Pz) and amplitudes (N2, P3) as the within-subject factors, and groups (SI vs. SA) as the between-subjects factors. The co-variants included age, sex, education, and BAI. An independent *t*-test was used to compare the go/no-go ERP amplitude and latency between the two groups. Additionally, a Pearson's correlation analysis was conducted between the go/no-go ERP and psychological measures. Differences were considered significant at *p* ≤ 0.05. All statistical analyses were performed using the SPSS version 26.0 (SPSS Inc., Chicago, IL, USA).

## Results

### Participants

Table 1 presents the demographic data and clinical measurements of all the patient groups. We classified 76 participants in the SI group and 74 in the SA group. No significant differences in age (*p* = 0.368), sex (*p* = 0.091), or educational level (*p* = 0.088) were observed between the two groups. The SI group had significantly higher BAI scores (*p* = 0.005) than the SA group. Meanwhile, the SA group had higher ACSS scores than the SI group (*p* = 0.002).

**Table 1:** Comparison of baseline demographic and clinical symptom characteristics between suicide ideators and suicide attempters.

	**Suicide ideators** **(*N* = 76)**	**Suicide attempters** **(*N* = 74)**	***p*-value**
	**Mean** **±SD or** ***N*** **(%)**		
Age (years)	39.38 ± 14.31	41.53 ± 14.78	0.368
Sex			
Male	30 (39.4)	31 (41.9)	0.091
Female	46 (60.6)	43 (58.1)	
Education (years)	11.97 ± 3.18	11.07 ± 3.27	0.088
Beck Depression Inventory (BDI)	53.63 ± 9.91	51.57 ± 13.37	0.284
Beck Anxiety Inventory (BAI)	32.68 ± 14.45	25.92 ± 14.78	**0.005**
Difficulties in Emotional Regulation Scale (DERS)	112.84 ± 25	106.28 ± 24.20	0.105
Barrett Impulsivity Scale (BIS)	74.45 ± 11.99	72.43 ± 9.97	0.265
Attention impulsivity	20.02 ± 3.95	18.76 ± 3.66	0.043
Motor impulsivity	25.22 ± 5.44	24.65 ± 4.39	0.477
Non-planning impulsivity	29.19 ± 5.05	29.03 ± 4.25	0.824
Acquired Capability for Suicide Scale (ACSS)	46.82 ± 16.72	54.84 ± 14.40	**0.002**

*Bold mean P-value < 0.05*.

### Behavioral Outcomes

Table 2 presents the behavioral outcomes of the no-go paradigm. No significant difference in behavioral outcomes was observed between the two groups.

**Table 2:** Comparison of behavioral outcomes between patients with suicide ideators and with suicide attempters.

	**Suicide ideators (*N* = 76)**	**Suicide attempters (*N* = 74)**	***p-*value**
Go accuracy (%)	95.20, 10.19	95.81, 10.90	0.720
No-go accuracy (%)	92.32, 9.94	91.73, 11.13	0.733
False alarm rate (%)	7.67, 9.94	8.27, 11.13	0.733
Reaction time (ms)	472.50, 95.40	469.70, 69.85	0.838

### ERP

#### Amplitude and Latency

**Figure 1:**
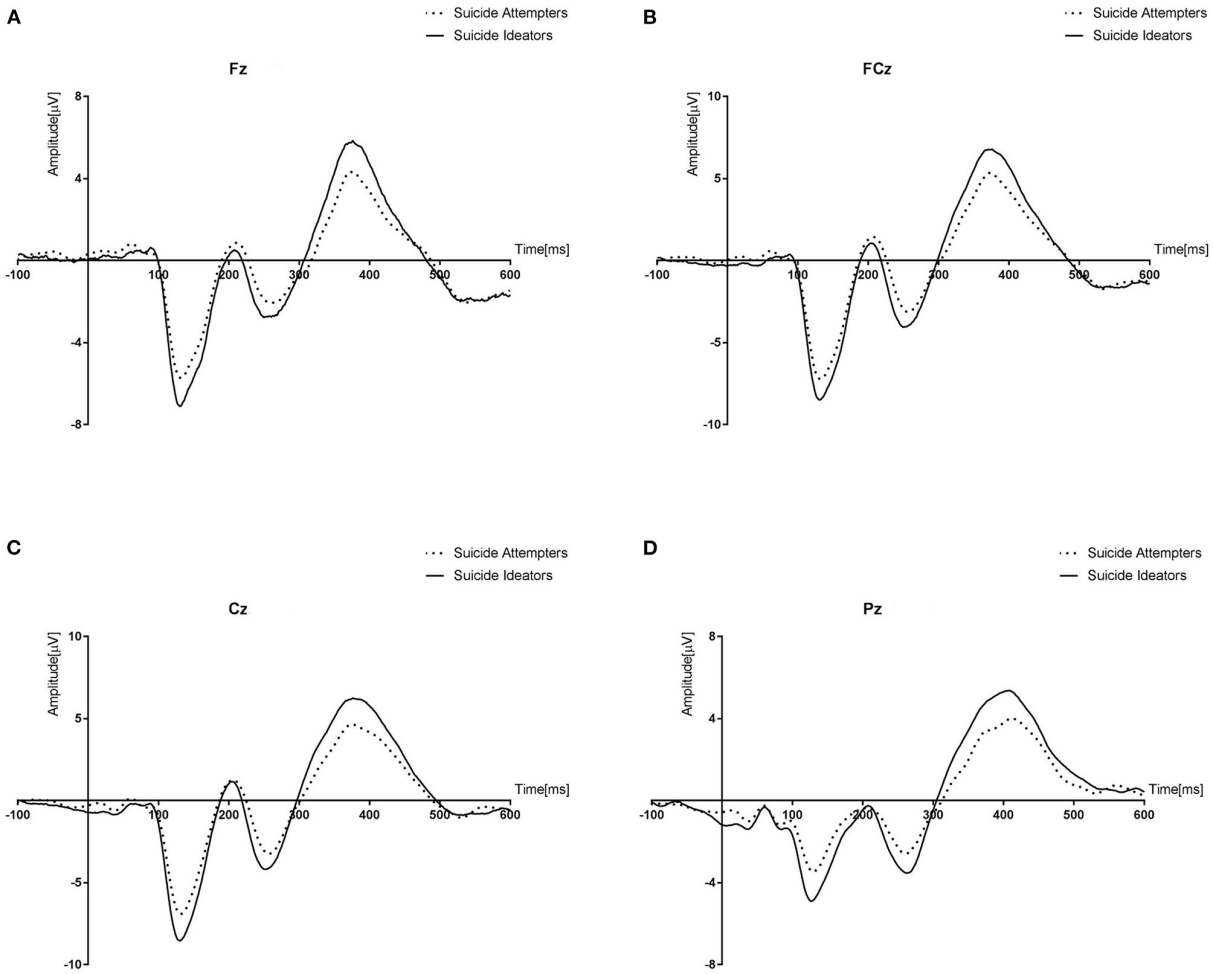
Grand averages of no-go Event Related potentials (ERPs) at all electrodes (Fz, FCz, Cz, and Pz) between suicide ideators and suicide attempters. Suicide ideators had more positive no-go P3 amplitude than those of suicide attempters at all electrodes. **(A)** no-go P3 amplitude at Fz. **(B)** no-go P3 amplitude at FCz. **(C)** no-go P3 amplitude at Cz. **(D)** no-go P3 amplitdue at Pz.

Table 3 presents the amplitude and latency data for no-go N2 and P3. In repeated measures ANOVA, a significant difference in amplitude was identified between the two groups in the test of the between-subjects effect (*p* ≤ 0.05). Figure 1 showed grand averages of no-go ERPs at all electrodes between suicide ideators and suicide attempters. Both groups demonstrated significant differences in the no-go P3 amplitude at all electrodes, which SI had more positive no-go P3 amplitudes than those of SA at all electrodes (Fz, *p* = 0.009; FCz, *p* = 0.023; Cz, *p* = 0.015; Pz, *p* = 0.001). However, no significant difference was observed in the no-go N2 amplitude.

**Table 3:** Comparison of the amplitude and latency of no-go N2, P3 between patients with suicide ideators and with suicide attempters.

**Site (μV)**	**Suicide ideators** **(*N* = 76)**	**Suicide attempters** **(*N* = 74)**	***t***	***p-*value**
	**Mean** **±SD or** ***N*** **(%)**			
**Amplitude**				
No-go FzN2	−5.39 ± 4.06	−4.25 ± 3.15	1.923	0.056
No-go FzP3	7.82 ± 5.06	5.97 ± 3.38	−2.639	**0.009**
No-go FCzN2	−5.60 ± 5.55	−5.00 ± 3.75	0.761	0.448
No-go FCzP3	8.91 ± 5.77	7.07 ± 3.89	−2.294	**0.023**
No-go CzN2	−5.96 ± 4.69	−4.92 ± 3.94	1.483	0.140
No-go CzP3	8.39 ± 5.36	6.54 ± 3.70	−2.461	**0.015**
No-go PzN2	−5.16 ±−3.15	−4.37 ± 2.77	1.636	0.104
No-go PzP3	7.25 ± 3.84	5.43 ± 3.00	−3.245	**0.001**
**Latency**				
No-go FzN2	262.37 ± 36.33	267.91 ± 36.01	0.937	0.350
No-go FzP3	383.08 ± 41.63	380.92 ± 37.60	−0.334	0.739
No-go FCzN2	258.96 ± 32.46	263.38 ± 27.81	0.896	0.372
No-go FCzP3	380.12 ± 41.10	375.01 ± 42.77	−0.754	0.452
No-go CzN2	257.63 ± 29.77	265.34 ± 31.02	1.552	0.123
No-go CzP3	384.53 ± 49.67	382.30 ± 49.82	−0.274	0.784
No-go PzN2	259.41 ± 31.82	259.20 ± 35.85	−0.037	0.971
No-go PzP3	409.50 ± 49.97	403.32 ± 55.34	−0.717	0.475

*Bold mean P value < 0.05*.

#### Correlations

**Figure 2:**
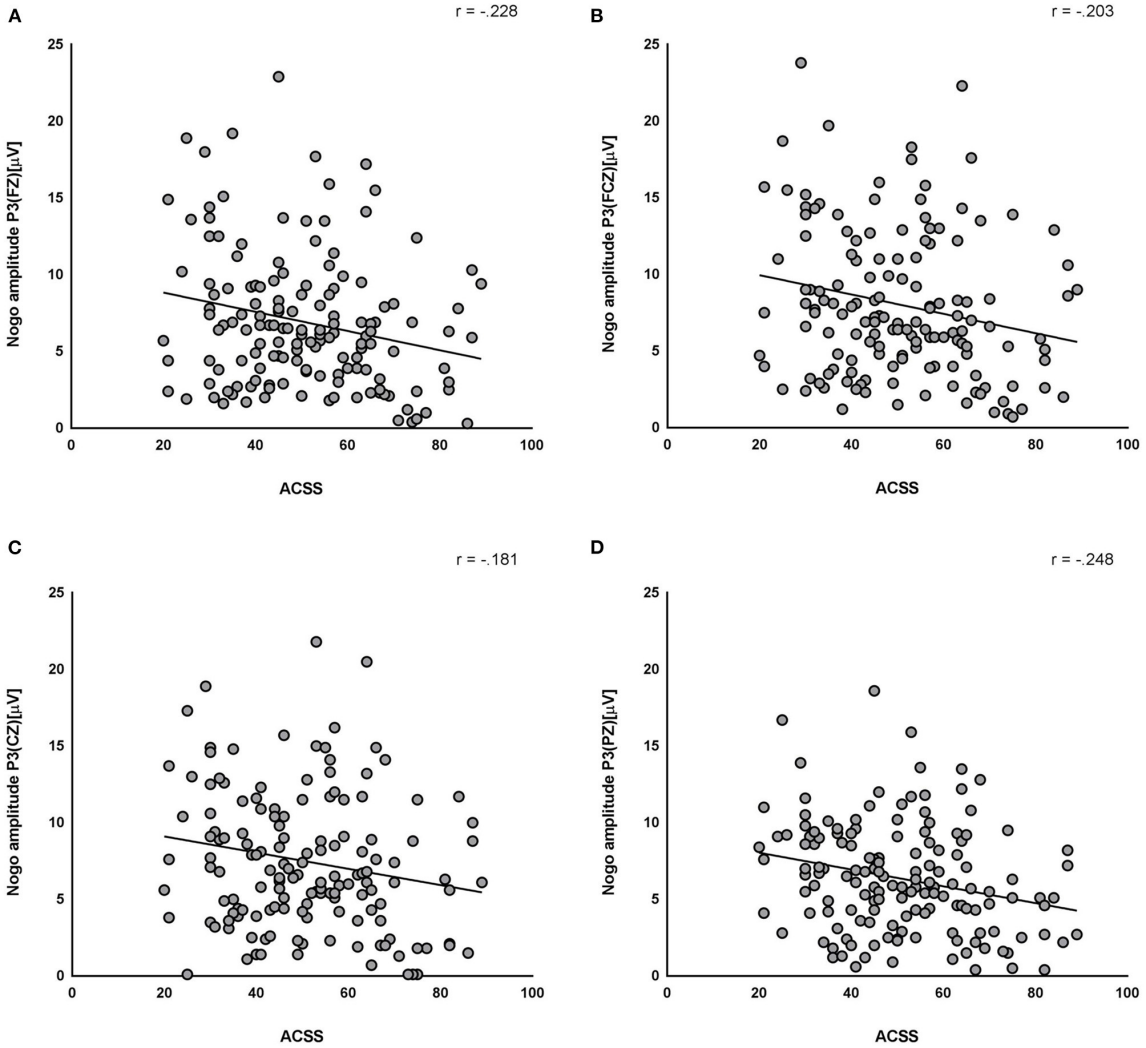
Correlations between the clinical measurements and Event Related Potentials (ERPs). Acquired Capability for Suicide Scale (ACSS) and no-go P3 at all electrodes (Fz: *r* = −0.228, *p* = 0.005; FCz: *r* = −0.203, *p* = 0.013; Cz: *r* = −0.181, *p* = 0.027; Pz: *r* = −0.248, *p* = 0.002) were negatively correlated. **(A)** no-go P3 at Fz and ACSS. **(B)** no-go P3 at FCz and ACSS. **(C)** no-go P3 at Cz and ACSS. **(D)** no-go P3 at Pz and ACSS.

Figure 2 presents the correlations between the clinical measurements and ERPs in all the participants. In the correlation analysis, no-go P3 amplitudes at all electrode sites were negatively correlated with the ACSS scores (Fz: *r* = −0.228, *p* = 0.005; FCz: *r* = −0.203, *p* = 0.013; Cz: *r* = −0.181, *p* = 0.027; Pz: *r* = −0.248, *p* = 0.002).

## Discussion

No-go ERP is known to be consistently related to impulsivity (21–23). As expected, there were significantly diminished no-go P3 amplitudes at all electrodes in the SA group, compared to the SI group, supporting the results of previous studies. Meanwhile, there was a negative correlation between the no-go P3 amplitude and ACSS, reflecting the suicide capability in our study. Unexpectedly, no significant difference in the BIS scores was identified between the two groups in this study.

A significant difference in the no-go ERP was identified in the SA and SI groups. The no-go P3 amplitudes of all electrodes measured in the SA group were significantly lower than those in the SI group. This finding is consistent with the results of previous studies in which the P3 amplitude decreased when implementing the no-go task in a group that had difficulties in controlling impulsivity (21–23). As reported in one meta-analysis, the no-go P3 ERP is known as a biomarker reflecting impulsivity (39), and no-go P3 is associated with direct control responses to inappropriate behavior during the response inhibition process (20, 40). Previous studies have also revealed that response inhibition control plays an important role in the progression from SI to SA as part of cognitive regulation related to the prefrontal cortex (6, 7). Moreover, considering that the no-go paradigm itself is related to impulsivity, and that P3 is an ERP component reflecting high cognitive function, the amplitude of P3 when performing the no-go task refers to the cognitive ability available to the subject when controlling impulsivity (41).

Although we expected that the SA group would have higher BIS scores than those of the SI groups, considering that previous studies have insisted on the association between impulsivity and suicide attempts (42), the SA group in the study did not differ from the SI groups in the BIS scores. There are several points of view regarding this result.

First, there may be a methodological problem in the measurement of impulsivity. The BIS-11 has a factor structure that purports to measure unique forms of impulsivity but is commonly used to assess a general unidimensional impulsivity construct (43). However, recent studies have failed to replicate this factor structure (43). Milner et al. (44) used the BIS-11 and UPPS-P to analyze the difference in impulsivity between the SA and SI groups to overcome these methodological limitations. In the above study, suicide attempters demonstrated higher negative urgency (one of the UPPS-P's impulsivity subdomains) scores than suicide ideators, with no significant difference in BIS-11 scores (44). Considering this result, Milner et al. (44) suggested that SA may be characterized by the propensity to reach higher levels of affect-related impulsivity, possibly only during particular circumstances or specific highly affective states that are directly related to attempting suicide. Future studies should evaluate the correlations between negative urgency and no-go ERPs.

Second, a recent study has suggested that differences in trait impulsivity between SI and SA (45) were insignificant or that impulsivity may be indirectly related to suicidal behaviors by potentially exacerbating more proximal risk factors (46, 47). Additionally, Neufield et al. suggested that trait impulsivity plays an important role as an additional risk factor and, in combination with hopelessness, has better predictive power for SI than hopelessness alone (48). In the context of the interpersonal theory of suicide (IPTS), the link between trait impulsivity and actual suicidal behavior has been explained by increased levels of capability for suicide due to different experiences because of differing levels of trait impulsivity (49). Our finding that the SA group had higher ACSS scores than the SI group supports the above hypothesis. Meanwhile, a different perspective can be gleaned from our findings. Hadzic et al. (50) found a significant correlation between trait impulsivity and suicidal capability. Hadzic et al. (50) has suggested that trait impulsivity may lead to capability for suicide, which is acquired over time, and could support the conceptualization of trait impulsivity as a distal risk factor for suicidal behavior. However, the different perspectives might be caused by the different characteristics of the study population (the time of SI and suicide lethality) and the different sample sizes.

Although the BIS-11 scores were not different between the two groups, the ACSS scores were significantly higher in the SA group than in the SI group in this study. Previous research has suggested that trait impulsivity is associated with higher levels of capability for suicide and thus leads to a higher risk of suicidal behavior (47). Venables et al. (9) suggested that low inhibitory control and high threat sensitivity could lead to emotional dysregulation, increasing suicide capability. Moreover, a negative correlation was identified between the no-go P3 amplitudes at all electrodes and the ACSS. Regarding the above relations, our results that the no-go P3 amplitudes are more decreased in the SA group than in the SI group might reflect the higher impulsivity of SA. Our results might be impossible to compare with those of previous studies because no study has evaluated the relationship between ACSS and no-go P3. However, regarding both no-go P3 and late positive potential (LPP) that could be observed in the ERP after the presentation of emotional stimuli (51, 52), one previous study that investigated the association between fearlessness of death, one subdomain of ACSS, and LPP might be considerable (53). Weinberg et al. (53) have reported that SA survivors had a blunted LPP to threatening images compared with controls in the SI group. This study provides evidence that the neural system that produces LPP is related to the capability for suicide. Moreover, evidence suggests that LPP is linked to a protracted orienting response, evident as a sustained P300. Hajcak et al. (54) have suggested that this orienting response is reflected in the LPP/P300 and may result from the phasic activity of the locus coeruleus (LC) norepinephrine system. Previous studies have identified an association between LC and SA (55–57). Gos et al. (56) have suggested that tyrosine hydroxylase immunoreactivity, which was associated with norepinephrine, was elevated in suicide victims who committed violent suicide and who could have higher self-aggression. Moreover, Pearlstein et al. (57) have suggested that persons with higher emotion-related impulsivity demonstrated a delay in response inhibition after increases in arousal (pupil), which could reflect LC function. Although LPP and go/no-go ERP are clearly different ERP components, the association between ACSS and no-go P3 in this study might reflect emotional dysregulation related to suicidal behaviors regarding the previous association of LPP with ACSS. In the future, studies on the association between ACSS, trait impulsivity, and other ERPs, such as LPP, are needed.

Our study had some limitations. First, we did not evaluate impulsivity in various aspects because we only used the BIS-11 to evaluate impulsivity. Future studies are needed to determine the association between suicide attempts and ERP using various impulsivity scales to evaluate subjects' multidimensional impulsivity (for example, UPPS-P). Second, since it was cross-sectional in nature, a longitudinal study would be needed to further evaluate the dynamic changes in serotonergic activity in the human brain. Third, various scales for measuring clinical characteristics have been evaluated using self-reported measures. Despite the self-reported scales in this study having good stability and validity, they were unable to reflect the neural/cognitive basis of the clinical characteristics such as impulsivity and emotional regulation. Fourth, our results may be generalizable to patients with major depressive disorder (MDD). Fifth, we did not consider the association between SI intensity and impulsivity. One study suggested that intensity of worst-point past SI was associated with impulsivity (58). Therefore, future studies should explore the relationship between SI intensity and impulsivity. Sixth, we did not consider other factors that could affect participants' ERP components such as early childhood trauma and obesity. Previous studies have suggested that early childhood trauma can affect a patient's inhibitory control, which could result in increased suicide risk (59, 60). Further, Wang et al. (61) suggest that the decreased effect of P3—not N2—might reflect the neural substrate of inhibitory control deficits in obese people. Therefore, future studies should attempt to exclude factors that may affect inhibitory controls like the abovementioned factors. Additionally, the past presence or absence of suicidal behavior and lifetime psychiatric diagnosis can also affect the ERP component. In practice, patients have various suicidal ideations—including plans to commit suicide and additional attempts after non-fatal suicide attempts—which is a common symptom of depression. However, there are difficulties with enrolling patients experiencing first time SI. Therefore, previous EEG or brain imaging studies which sought to classify and distinguish SI from SA did not consider patients' past psychiatric diagnoses, SA, and SI history (5, 25, 62–65). Thus, we attempted to exclude this confounding variable, we enrolled drug naïve participants and included them in the SA or SI groups. Future studies should consider psychiatric diagnoses or SA history to strengthen the current literature. Finally, in this study, the clinical characteristics in SI and SA were not compared with a normal control group. Hadzic et al. (50) reported no significant association between trait impulsivity and SI. However, another study reported a significant association between SI and impulsivity (24, 48). Considering this inconsistent relationship between impulsivity and suicidal ideation in the literature, future studies will need to compare impulsivity in normal controls, suicide attempters, and suicide ideators.

This study was able to compare no-go ERPs between patients with MDD who had SA and SI. Moreover, we also evaluated ERPs immediately after near fatal SA in a relatively large, drug naïve sample. Hence, the attenuated no-go P300 amplitude might reflect SA in patients with MDD. Future studies should explore the various aspects of impulsivity in suicide which may be related to no-go ERP.

## Data Availability Statement

The datasets presented in this article are not readily available due to ethical and privacy concerns. Requests to access the datasets should be directed to JK, ideal91@hanmail.net.

## Ethics Statement

The studies involving human participants were reviewed and approved by the Institutional Review Board and Ethics Committee of Soonchunhyang University Cheonan Hospital. The patients/participants provided their written informed consent to participate in this study.

## Author Contributions

JK has full access to all of the data in the study and take responsibility for the integrity of the data and the accuracy of the data analysis. SY and JK conceived of the study, participated in its design, coordination, and drafted the manuscript. SY performed the data acquisition, sampling, and performed the data analysis. All authors approved the final version and all take responsibility for its content.

## Funding

This work has supported by the Basic Science Research Program through the National Research Foundation of Korea (NRF) funded by the Ministry of Education (2020R1-1A3A04036435) and (2020R1-1A3068017).

## Conflict of Interest

The authors declare that the research was conducted in the absence of any commercial or financial relationships that could be construed as a potential conflict of interest.

## Publisher's Note

All claims expressed in this article are solely those of the authors and do not necessarily represent those of their affiliated organizations, or those of the publisher, the editors and the reviewers. Any product that may be evaluated in this article, or claim that may be made by its manufacturer, is not guaranteed or endorsed by the publisher.

## Abbreviations

ACSS: acquired capability for suicide scale
ANOVA: analysis of variance
BAI: beck anxiety inventory
BDI: beck depression inventory
Cz: central
DERS: difficulties in emotion regulation scale
ERP: event-related potential
FCz: fronto-central
FPz: frontoparietal
IPTS: interpersonal theory of suicide
K-BIS-11-R: korean version of barratt impulsiveness scale-11-revised
LC: locus coeruleus
LPP: late positive potential
Pz: parietal
SA: suicide attempts
SI: suicidal ideation.
