# Corrigendum: Electrophysiological changes between patients with suicidal ideation and suicide attempts: An event-related potential study

**DOI:** 10.3389/fpsyt.2022.969450

**Published:** 2022-08-24

**Authors:** Sung Hoon Yoon, Se-Hoon Shim, Ji Sun Kim

**Affiliations:** Department of Psychiatry, Soonchunhyang University Cheonan Hospital, Cheonan, South Korea

**Keywords:** impulsivity, suicidal ideation, suicide attempt, ERP, ACSS

In the published article, there was an error in the Acknowledgements. Soonchunhyang University Cheonan Hospital was incorrectly acknowledged. The correct statement appears below.

The authors apologize for this error and state that this does not change the scientific conclusions of the article in any way. The original article has been updated.

## Acknowledgments

This study was also supported by Soonchunhyang University.

## Publisher's note

All claims expressed in this article are solely those of the authors and do not necessarily represent those of their affiliated organizations, or those of the publisher, the editors and the reviewers. Any product that may be evaluated in this article, or claim that may be made by its manufacturer, is not guaranteed or endorsed by the publisher.

